# Heterosexual Anal Sex among Female Sex Workers in High HIV Prevalence States of India: Need for Comprehensive Intervention

**DOI:** 10.1371/journal.pone.0088858

**Published:** 2014-02-20

**Authors:** Mallika Alexander, Mandar Mainkar, Sucheta Deshpande, Shweta Chidrawar, Suvarna Sane, Sanjay Mehendale

**Affiliations:** 1 Clinical Trials Unit, National AIDS Research Institute, Pune, Maharashtra, India; 2 Integrated Behavioural and Biological Assessment Project, National AIDS Research Institute, Pune, Maharashtra, India; 3 Department of Biostatistics and Epidemiology, National AIDS Research Institute, Pune, Maharashtra, India; 4 National Institute of Epidemiology, Chennai, Tamil Nadu, India; UCL Institute of Child Health, University College London, United Kingdom

## Abstract

**Introduction:**

Role of vaginal sex in heterosexual transmission of HIV has been investigated but that of heterosexual anal sex (HAS) is not fully understood. This paper examines practice of HAS among Female Sex Workers (FSWs) and its correlates in India where the HIV epidemic is being primarily driven by core groups like FSWs.

**Methods:**

Data for this paper are drawn from Round I survey of 9667 FSWs in the Integrated Biological and Behavioral Assessment (IBBA) from 23 districts of 4 high HIV prevalent states of India. Bivariate and multivariate analysis identified factors associated with HAS.

**Results:**

Ever having anal sex was reported by 11.9% FSWs (95% CI: 11.3%–12.6%). Typology (AOR 2.20, 95% CI 1.64–2.95) and literacy (AOR 1.28, 95% CI 1.10–1.49) were positively associated with practice of HAS. Longer duration in sex trade (AOR 1.69, 95% CI 1.44–1.99), entertaining larger number of clients the previous week (AOR 1.78, 95% CI 1.47–2.15), alcohol consumption (AOR 1.21, 95% CI 1.03–1.42) and inability to negotiate condom use (AOR 1.53, 95% CI 1.28–1.83) were also correlated with HAS. Self-risk perception for HIV (AOR 1.46, 95% CI 1.25–1.71) did not impede HAS. Although symptoms of sexually transmitted infections (STIs) in the last 12 months were associated with anal sex (AOR 1.39, 95% CI 1.13–1.72) there was no significant association between laboratory confirmed HIV and other STIs with HAS.

**Conclusion:**

Practice of HAS by FSWs might significantly contribute to HIV transmission in India. This study also shows that despite self-risk perception for HIV, even literate FSWs with longer duration in sex work report HAS. General messages on condom use may not influence safe HAS. FSWs need to be targeted with specific messages on HIV transmission during anal sex. Women controlled prevention methods, such as rectal microbicides and vaginal microbicides are needed.

## Background

It has been estimated that nearly 2.1 million people are presently living with HIV in India. [1], [2] Men who buy sex and their female partners constitute the largest group of people living with HIV in India and Asia. [1] The national behavioral surveillance has reported that three percent of sexually active Indian males have had sex with a female commercial sex worker in the last one year. [3] This supports the fact that sex work is an important driver of the HIV epidemic in India. However programs implemented by National AIDS Control Organization of India promoting safe sex have resulted in reduction of prevalence of HIV among FSWs from 5.06% in 2007 to 2.67% in 2011. [1], [2] Global data suggest that at least 10% women in heterosexual relationships practice anal sex. [4], [5], [6], [7], [8] But very little attention has been given to the role of heterosexual anal sex (HAS) in transmission of HIV to women even though evidence through systematic review shows that HIV risk associated with receptive anal intercourse is almost eight times higher even if the infected partner is receiving HAART. [4], [9], [10], [11] Meta-analysis of studies from developed countries has shown that probability of HIV transmission is higher per act of receptive anal sex (1.7%) as compared to peno-vaginal sex (0.8%). [12] It has been estimated that among heterosexual couples practicing anal sex, the cumulative risk of HIV acquisition within a period of three months can increase up to nine times in the women if two out of their eight encounters involve anal sex. [13] Associated risk behaviors innate to sex trade such as substance use and multiple partners augment the risk of anal sex. [14], [15] Motivation for engaging in unprotected HAS in FSWs could be more related to their occupation instead of factors such as intimacy, physical pleasure, experimentation, etc. cited by other heterosexual couples. [14], [16], [17], [18] Studies have documented that several high-risk behaviors such as sex work, multi-partner sex, anal sex and substance abuse occur together. [19] Therefore heterosexual anal sex could be considered a proxy for overall high-risk sexual behavior. Condom use is universally reported to be lower during HAS than vaginal sex. [10], [20] Available data from recent studies report high prevalence and lack of condom use during anal sex. [3], [14] However, there is lack of information on HIV transmission risk due to unprotected heterosexual anal sex in high-risk populations in India. FSWs have a higher risk of HIV acquisition from HIV infected clients demanding anal sex and once infected they can transmit HIV infections to other clients especially if they are not able to negotiate condom use. Anal sex offered by FSWs in India might be contributing significantly to HIV transmission, but its role needs to be investigated. There is a need to cause further reduction in HIV transmission from core groups like FSWs to the general population through the bridge population of clients of sex workers. For this, it is important to identify risk factors associated with HAS and implement appropriate preventive strategies through the national program.

With this background of scarce data on practice and predictors of HAS among FSWs in India, this paper reports the extent of practice of HAS in FSW population in India and discusses the risk factors and predictors independently associated with this practice.

## Methodology

### Ethics Statement

ICMR institutes- The National AIDS Research Institute, (NARI) Pune, National Institute of Epidemiology, Chennai, National Institute of Nutrition, Hyderabad, and Karnataka Health Promotion Trust, Bangalore, implemented the IBBA in Maharashtra, Tamil Nadu, Andhra Pradesh and Karnataka states respectively. Ethical approvals for this primary IBBA survey were obtained, prior to the survey from the Protection of Human Subjects Committee of Family Health International and the ethical committees of all participating institutes mentioned above. Written consents were obtained from all respondents before conducting the interview, physical examination and collection of blood and urine samples. The survey was conducted anonymously and no names or personal identifiers were linked to data sheets. All consented respondents were given a unique identification number. One to one interviews were conducted in the vicinity of the cruising points by trained interviewers. Extensive training of the interviewers was conducted to ensure appropriate fielding of sensitive questions, sensitization to various sex practices, confidentiality, non-judgmental approach, etc. Other protective measures followed by the investigators included oaths of confidentiality by all survey staff, development of harm minimization guidelines and specimen and data safety guidelines. Behavioral and biological information was linked anonymously to safeguard the participant’s right to confidentiality. Services such as STI examination, syphilis treatment, referral to STI clinics and voluntary counseling and testing (VCT) were offered to the respondents. Monetary compensation generally equivalent to a day’s worth of wages was provided to the respondents. In addition to these measures, Community Monitoring and Advisory Boards were set up in the surveyed districts to oversee ethical conduct of the survey.

Data for this paper are drawn from the Round I survey of IBBA involving 9667 FSW from 23 districts in four high HIV prevalence states of India, namely, Tamil Nadu, Andhra Pradesh, Karnataka and Maharashtra. The districts for IBBA were chosen purposively, based on each state’s socio-cultural regions and size of the FSW population. The first round of IBBA was conducted over a 19-month period between November 2005 and June 2007. [21], [22] This survey was intended to serve as a baseline for the impact evaluation of the Avahan India AIDS initiative, a large HIV prevention program supported by the Bill and Melinda Gates Foundation. These data uniquely capturing behavioral as well as biological data on core risk groups for HIV transmission in India are available for analysis and interpretation. [Data access - http://www.nari-icmr.res.in/IBBAdataaccess.php].

### Sampling

A pre-survey assessment with district-wide mapping of the core group sites helped to define survey sub-groups, establish methodology and decide the sampling procedures. Information on sites of congregation of FSWs, hours of operation and an estimate of the number of eligible respondents available at different times of the day and on different days of the week was collected and analyzed for development of the sampling frame.

### FSW Typology

In the original IBBA round 1 survey, FSW were defined as women aged 18 years or older who had exchanged sex for money at least once in the past one month. Operational definitions and eligibility criteria have been described in the earlier publications. [21], [22] The typology of FSW was decided based on their solicitation point of sex work such as brothels, streets, lodges and homes. Non-brothel based FSWs included those who operated from streets, lodges and homes.

### Sample Size

The sample size of 400 for each survey sub group (typology) per district allowed for the detection of an absolute difference of 15% or more of high-risk behavior from the assumed value of 50%, with 95% confidence and 90% power. A design effect of 1.7 was assumed for cluster sampling, based on the best available information at that time. Response rate was 79.8% (min-max: 69.1–89.9%).

In Mumbai, Thane and Pune brothel based and non brothel based FSWs were separately sampled. In the remaining 20 out of 23 selected districts where the FSW population was less than the required sample size for a specific typology, respondents were selected from both brothel and non brothel based typologies to meet the sample size target of 400 for that district (combined group).

### Sampling Procedure

Conventional cluster sampling was used for brothel-based and home-based sex workers. Time Location Sampling (TLS) was used for non-brothel-based FSW other than home based FSWs. Selection of respondents for conventional cluster sampling/time-location cluster sampling was done through a two-stage cluster sampling procedure. The primary sampling units (clusters) were selected by systematic random sampling (without replacement), by probability proportional to size. In the selected clusters, quick listing was done by unique identifier information rather than by name. All individuals who visited the site during the field timing were listed for time location sampling. Survey respondents were selected randomly using their dress code as labels from all eligible respondents available during the fixed time intervals. For a conventional cluster sample, all individuals affiliated with the site who met the eligibility criteria were listed, even if they were not present at the time of the field visit. A unique ID number was given to each of the listed individuals. Home-based sex workers were enumerated with the help of community liaison person at the selected cluster without explicitly identifying the eligible respondents. For example, houses were given numbers or unique identifiers such as, blue painted wall (house no. 2), balloon like lamp at the front door of house (house no. 5), etc. Eligible respondents from the specific clusters were then selected randomly to meet the required numbers. Study supervisors were generally responsible for listing, selecting and approaching respondents for recruitment with the help of a community liaison.

For populations that did not congregate at identifiable locations, where insufficient proportion of members was accessible to the interviewers to represent the larger group and where the population was sufficiently networked, Respondent Driven Sampling (RDS) was used. However data from 1108 FSWs collected through RDS from bar-based FSWs and certain other FSW populations in Mumbai and Parbhani districts in Maharashtra were excluded from the present analysis, as it is not generally possible to directly compute the sampling weights necessary for traditional design-based inference.

### Behavioral Measures

The questionnaire was developed in English by experts from the sponsoring organizations, participating institutes and authorities on the subjects from India and other countries. It was translated into local languages and back translated into English, to ensure accuracy and uniformity. Pilot testing of the questionnaire was carried out prior to the actual survey. Efforts were taken to include locally used terminologies for various sexual acts.

The dependent variable of ever having had anal sex was measured by dichotomous response of “yes” or “no” to the question ‘‘Have you ever had anal intercourse with a client?” Independent variables included socio demographic variables such as literacy, source of income, FSW typology and place of entertaining clients. Response to question on marital status included “ever-married” and “unmarried”. Hence ever-married category includes those who were currently married as well as those who were widowed, separated, deserted and divorced. Sex work profile was assessed through number of days and number of clients entertained in the previous week and number of years in sex work. Knowledge and awareness on STI, HIV and self-risk perception of HIV acquisition among FSWs were assessed with a dichotomous response of “yes” or “no”. Risky behavior was measured by condom use at last sex with a paying partner. Data on inability to negotiate condom use, condom breakage, alcohol consumption and forced sex experience of FSW were also obtained. Self reported symptoms of STI were noted. Detailed description of the behavioral measures has been provided in Table 1.

**Table 1:** Behavioural measures description.

Variables	Question	Measure
Ever had anal sex	Have you ever had anal intercourse with a client?”	Dichotomous response of “yes” or “no”.
FSW subgroups (Typology)	Completed by supervisors based on sampling framedeveloped from mapped physical locationsof solicitation points where FSWscongregate.	Brothel based-Solicits from brothel. Non brothel based- Solicits from street, home, lodges, etc. Non brothel based- Solicits from street, home, lodges, etc.Combined-In districts where the number of FSWs were less than the required sample size of 400 in the brothels or non brothel site of solicitation
Place of entertaining clients	Where do you generally entertain most of your clients”?	Responses were recorded as brothel and non brothel based
Literacy	Can you read and write?	Responses recoded as illiterate for those who did not have any education and literate for those who could read only and those who could read and write
Marital status	Have you ever been married?	Dichotomous response of “yes” or “no”. Yes was considered as ever married and no as unmarried. Ever married status included those who were currently married as well as those who were widowed, separated, deserted and divorced.
Source of income	Apart from sex work, what is your main source of income?	Captured responses were recorded to none and have othersource of income.
Number of days entertained clientsin the previous week	How many days did you have sexual intercoursewith clients in the **past** **week** (7 days)?	Continuous responses were recorded as 1 to 3 and 4 to 7 for the former.
Number of clients entertained inthe previous week	Can you tell me, how many clients did you havesexual intercourse with in the**past week** (7 days)?	Continuous responses were recorded 1 to 7 and more than 7 for the latter variable.
Number of years at sex work	a. How old are you now ?b. How old were youwhen you started sex work*?*	Continuous variable of number of years at sex work was derived by subtracting age at first paid sex from the reported current age. This was further recoded as “upto 3 years” and “more than 3 years”.
Awareness of STI	Have you ever heard of diseases that can be transmittedthrough sexual intercourse?	Dichotomous response of “yes” or “no”.
HIV and self risk perception ofHIV acquisition	Do you yourself feel you are at risk to be infectedwith HIV/AIDS?	Dichotomous response of “yes” or “no”.
Condom use at the last sex withpaying partner	a. The **last time** you had sexual intercourse withan **occasional client**, did he use a condom? b The**last time** you had sexual intercourse with a**regular client**, did he use a condom?	A variable was computed that coded use of condom at last sex with a regular paying partner and with occasional paying partner as “yes” and “no”.
Condom use at last anal sex	The **last time** you had anal intercourse witha client did he use a condom?	Dichotomous response of “yes” or “no”.
Inability to negotiate condomuse-Past month	In the **past month** was there a time when you wantedto use a condom with a client but didnot use it?	Dichotomous response of “yes” or “no”.
Condom breakage- past month	In the **past month**, have you had the experienceof a condom breaking while it wasbeing used?	Dichotomous response of “yes” or “no”.
Alcohol consumption- past month	During the past month, how often have youconsumed drinks containing alcohol?	Responses were recorded as “yes” if consumed every day or atleast once a week and “no” for the rest of the responses.
Forced sex experience- past oneyear	In the **past one year**, were you ever beaten or otherwisephysically forced to have sexual intercourse withsomeone even though you didn’twant to?	Dichotomous response of “yes” or “no”.
Self reported symptoms of STIpast 12 months	a. During the past 12 months have you suffered fromlower abdominal pain without diarrhea or menses?b. During the past 12 months have you suffered fromvaginal discharge? c. During the past 12 months have yousuffered from genital ulcers or sores?	If the respondent reported yes to any one of these questions, she was considered to have STI symptoms in the last 12 months.
Current STI symptoms	Do you have any of the following **AT PRESENT?**	If the respondent reported presence of any one of these symptoms: burning on urination or foul-smelling vaginal discharge or genital ulcer/sore or swelling in groin area or any other genital complaints, it was coded as having STI symptom at the time of survey.

### Biological Measures

Blood, urine and genital ulcer swabs (only from the FSWs who consented for physical examination) were collected at the interview site and transported to the testing laboratories duly maintaining the cold chain. All quality control tests were performed at National AIDS Research Institute [NARI] on 10% of randomly selected sera, all N. gonorrhoea and C. trachomatis positive urine samples and 5% randomly selected negative urine samples. The proficiency of the laboratories was monitored using a structured quality assessment scheme. Results of syphilis serology (Rapid Plasma Reagin: RPR), confirmatory syphilis testing (Treponema Pallidum Haemagglutination Assay: TPHA), Human Simplex Virus (HSV-2: antibody EIA), HIV (antibody EIA), N. gonorrhoeae and C. trachomatis (APTIMA nucleic acid amplification) are presented in this paper.

Supervision and quality control measures implemented ensured quality of the data collected. All data were entered twice using CSPro (version 3.1), reconciled, cleaned, merged and weighted. More details of the survey design, steps in implementation and details of weighting process in IBBA can be found in earlier publications [21], [22], [23].

### Statistical Analysis

The data were analyzed using SPSS version 15.0. For this paper, the primary data were analyzed to estimate prevalence of HAS in the FSW population with a confidence interval of 95%. We performed cross-tabulation and logistic regression using alpha = 0.05 for significance testing. Analysis was weighted to account for differential recruitment of FSW within districts, differential non-response rates and differential probabilities of selection across districts and states. There were about 11% missing data for the outcome variable and the cases with the missing outcome data were omitted from the analysis. The paper presents data on (a) prevalence of anal sex among FSWs from four states of India and (b) correlates of anal sex. Factors reported in literature to be associated with heterosexual anal sex and other risky behaviors of FSW population in literature were included in bivariate analysis. Multivariate regression models included variables that were found to be significantly different among FSWs reporting HAS in the bivariate models as well as those that have been reported in the literature. The multivariate model adjusted for literacy and number of years in sex work examined the relationship between predictive variables on the outcome variable of HAS. Since several independent variables were being used in the regression model, collinearity and multicollinearity were ruled out by regressing each independent variable against the others. Interactions were also examined for scientifically relevant variables.

## Results

### FSW Profile

Selected information on socio-demographic and behavioral characteristics of the 9667 FSWs is summarized in Table 2. Median age of the FSWs was 30 years (range 18–60 years min-max). Nearly 60% were illiterate and among those who were educated, median number of years of education was 7 years (range 1–15 years min-max). In all, 84.5% were ever married including those who were currently married as well as those who were widowed, separated, deserted and divorced. Nearly half of the FSWs (47.8%) had no source of income other than sex work. Almost 20% reported to have initiated paid sex when they were less than 20 years of age. Also 65% of FSWs solicited paid sex from streets and public places. Median number of paying clients they had sex with, on the previous day was 2 (range 0–25 min-max) and that for the previous week was 7 (range 0–85 min-max). Almost three in ten FSWs (29%) reported not to have used condom at the last sex with their regular paying clients. Of the surveyed FSWs, 70% reported having non-paying partners. Nearly 70% of them never used condoms with their non-paying partners in the previous week. Alcohol consumption at least once a week in the last month was reported by 35%. More than 90% had heard of HIV (not shown in the table) and 83% were aware of STI. Presence of at least one of the four STI symptoms (genital ulcer, burning micturition, swelling in groin or vaginal discharge) was reported by 47.5% of the FSWs in the previous year and by 29% at the time of survey.

**Table 2:** Socio demographic and sex work profile of FSWs.

Characteristics	Total FSWs = 9667
Age - median (min, max)	30.0 (18, 60)
Age<25 years	18.30%
Age≥25 years	81.70%
No. of years of education, Median (min, max):	7.0 (1, 15)
Cannot read/write	60.30%
Can read only	11.00%
Can read and write	28.70%
Ever married	84.50%
Have no other source of income	47.80%
Age at initiation of sex <15 yrs	24.40%
Age at initiation of sex ≥15 yrs	75.60%
Age at first paid sex <20 yrs	19.10%
Age at first paid sex ≥20 yrs	80.90%
Solicits from brothel/lodge/dhabha	17.00%
Solicits from home	17.40%
Solicits from public places, others	65.30%
No. of clients FSW had sex with the previous day, Median(min, max):	2.0 (0, 25)
No. of clients FSW had sex with the previous week Median(min, max):	7.0 (0, 85)
Have regular paying clients	92.10%
Have occasional paying client	90.30%
Have non-paying partner	70.20%
Never used condom with non paying partners- last week	70.40%
Condom non use at last sex with regular client	29.00%
Have >2 types of partners	61.30%
Alcohol consumption at least once a week in the last month	34.80%
STI awareness	82.70%
STI at least one symptom-past 12 months	47.50%
STI at least one symptom-current	28.70%

### STI Prevalence

HIV prevalence among the study respondents was 14.1%. Almost half of the 2804 FSWs tested for TPHA were positive confirming that they had syphilis infection in the past. Less than 5% tested positive for chlamydial and gonococcal infections by Nucleic Acid Amplification Test [NGCT NAAT]. Of the 2816 tested for HSV-2 antibodies, 68% were positive (Table 3).

**Table 3:** Laboratory confirmed prevalence of STI.

STI	% (n = 9667)
HIV	14.1
RPR	11.6
TPHA*(n = 2804)	47.3
CT	4.0
NG	2.4
HSV-2*(n = 2816)	67.8

*of those who were tested.

HIV: Human Immunodeficiency Virus. RPR: Rapid Plasma Reagin for syphilis.

TPHA: Treponema pallidum HemoAgglutination confirmatory test for syphilis.

CT: Chlamydia trachomatis. NG: Neiserria gonorrhoea. HSV-2: Human Simplex Virus-2.

### Results of Bivariate Analysis

**Figure 1:**
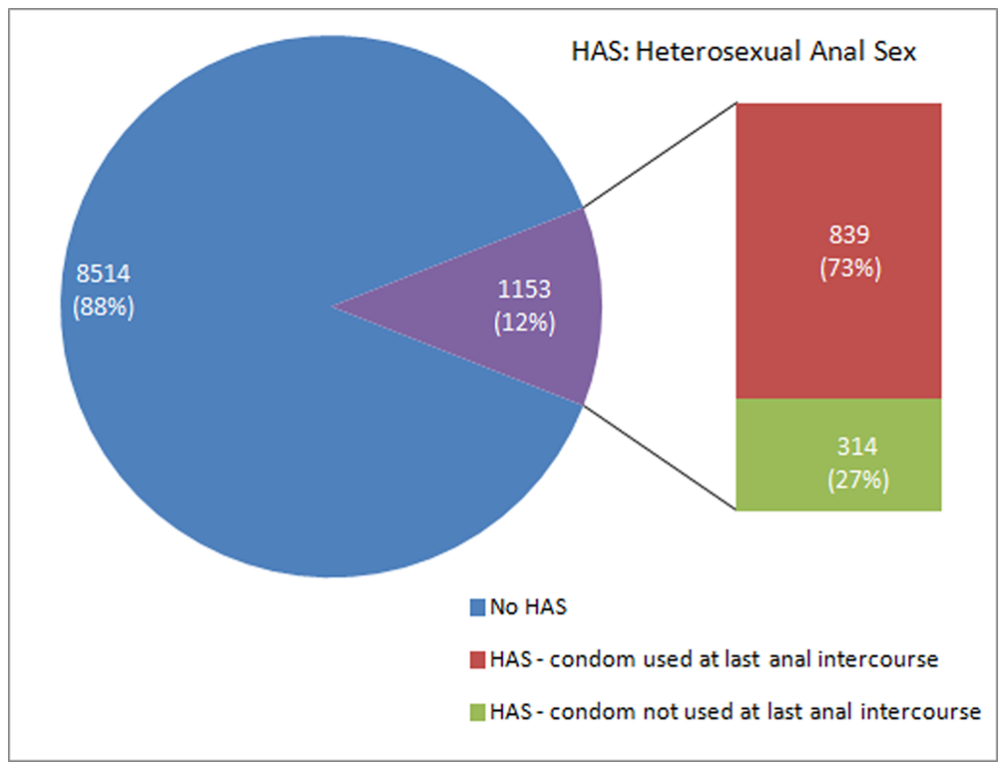
FSWs Heterosexual Anal Sex & condom used at last anal sex.

Hetero-sexual Anal Sex (HAS) was reported by 11.9% of the FSWs and of these 73% reported condom use at the last HAS (Figure 1). Table 4 shows the differences in characteristics of FSWs who reported HAS and those who did not. Significantly higher proportion of combined typology FSWs as compared to brothel based FSWs (OR 3.23, 95% CI 2.57–4.06) reported HAS. It was observed that FSWs entertaining clients at sites other than brothels (OR 1.84, 95% CI 1.51–2.24) and those who were in the sex trade for longer duration (OR 2.00, 95% CI 1.76–2.29) were more likely to report HAS. Significantly higher proportion of FSWs who were ever married (OR 1.43, 95% CI 1.19–1.73) and were literate (OR 1.16, 95% CI 1.02–1.31) were more likely to report HAS. Similarly those who had engaged in sex work for more than 3 days in the previous week (OR 1.15, 95% CI 1.01–1.30) and those who had entertained more than 7 clients in the previous week (OR 1.58, 95% CI 1.39–1.79) were also more likely to report HAS. Whereas FSWs who had no source of income other than commercial sex work (OR 0.65, 95% CI 0.58–0.74) were significantly less likely to report HAS.

**Table 4:** Bivariate and multivariate analysis for heterosexual anal sex: (logistic regression model using global weights).

Variables	n	Anal sex Yes#	%	OR* (95% CI)	p value	AOR* (95% CI)	p value
FSW sub-group							
Brothel based	1497	87	5.8	ref		ref	
Non brothel based	1182	76	6.4	1.12 (0.81–1.53)	0.51	0.77(0.53–1.13)	0.18
Combined (brothel and non brothel)	5963	990	16.6	3.23 (2.57–4.06)	**<0.001**	2.20(1.64–2.95)	**<0.001**
Place of entertaining client							
Brothel-only	1459	122	8.4	ref		Ref	
Non brothel-only	7176	1030	14.4	1.84 (1.51–2.24)	**<0.001**	1.21(0.93–1.58)	0.17
Current age							
<25 years	1548	195	12.6	ref			
≥25 years	7085	958	13.5	1.10 (0.98–1.31)	0.34	NA	NA
Marital status							
Unmarried	1362	139	10.2	ref		ref	
Ever married	7197	1004	14	1.43 (1.19–1.73)	**<0.001**	1.02(0.80–1.29)	0.88
Literacy level							
Illiterate	5075	641	12.6	ref		Ref	
Literate	3564	512	14.4	1.16 (1.02–1.31)	**0.02**	1.28 (1.10–1.49)	**0.002**
Source of income other than sex work							
Other source	4365	688	15.8	ref		Ref	
None	4232	460	10.9	0.65 (0.58–0.74)	**<0.001**	0.90(0.77–1.05)	0.17
Years in sex work							
Upto 3 years	3984	367	9.2	ref		ref	
>3 years	4648	786	16.9	2.00 (1.76–2.29)	**<0.001**	1.69 (1.44–1.99)	**<0.001**
Days entertained clients last week							
1 to 3	3634	456	12.5	ref		Ref	
4 to 7	4923	697	14.2	1.15 (1.01–1.30)	**0.031**	0.78(0.64–0.94)	**0.01**
Number of clients entertained last week							
1 to 7	4721	524	11.1	ref		Ref	
>7	3781	623	16.5	1.58 (1.39–1.79)	**<0.001**	1.78(1.47–2.15)	**<0.001**
Condom use at last sex with paying partner							
No	2701	394	14.6	ref		ref	
Yes	5941	759	12.8	0.85 (0.75–0.96)	**0.02**	0.97(0.82–1.14)	0.71
STI awareness							
No	1447	82	5.7	ref		ref	
Yes	7142	1060	14.8	2.68 (2.13–3.30)	**<0.01**	1.23(0.91–1.66)	0.18
Self risk awareness of HIV acquisition							
No	4428	467	10.5	ref		ref	
Yes	2434	506	20.8	2.23 (1.94–2.55)	**<0.01**	1.46(1.25–1.71)	**<0.001**
Wanted to use condom but couldn’t-last month							
No	6575	695	10.6	ref		Ref	
Yes	2003	438	21.9	2.37 (2.08–2.71)	**<0.001**	1.53(1.28–1.83)	**<0.001**
Condom breakage-Last month							
No	6932	705	10.2	ref		Ref	
Yes	1485	405	27.3	3.32 (2.89–3.81)	**<0.01**	2.03(1.70–2.42)	**<0.001**
Frequent consumption of alcohol-last one month							
No	5802	601	10.4	ref		Ref	
Yes (everyday/once a week)	2837	552	19.5	2.09 (1.85–2.37)	**<0.01**	1.21(1.03–1.42)	**0.02**
Forced sex-Last 12 months							
No	7447	757	10.2	ref		ref	
Yes	1179	390	33.1	4.36 (3.78–5.03)	**<0.01**	2.24(1.87–2.67)	**<0.001**
STI symptoms-Last 12 months							
No	4717	327	6.9	ref		Ref	
Yes	3925	826	21	3.58 (3.12–4.10)	**<0.001**	1.39 (1.13–1.72)	**0.002**
STI symptom-current							
No	6304	559	8.9	ref		Ref	
Yes	2338	594	25.4	3.50 (3.08–3.98)	**<0.001**	1.59 (1.30–1.94)	**<0.001**

*OR-Odds Ratio; AOR-Adjusted Odds Ratio (Adjusted for literacy and years at sex work).

# Denominators will change as per the distribution of missing data/non-responses for anal sex in categories. Percentages are calculated taking the appropriate denominator.

FSWs who used condom at the last sex with their paying partner were significantly less likely to report HAS (OR 0.85, 95% CI 0.75–0.96). Whereas those who were aware of STIs (OR 2.68, 95% CI 2.13–3.30) and also of their own risk of HIV acquisition (OR 2.23, 95% CI 1.94–2.55) had higher odds of reporting HAS than those who were unaware. Bivariate analysis of risk profile of FSWs shows that factors such as inability to negotiate condom use with their clients in the previous month (OR 2.37, 95% CI 2.08–2.71), experience of condom breakage in the last month (OR 3.32, 95% CI 2.89–3.81) were associated with HAS. Furthermore consumption of alcohol at least once a week in the previous month (OR 2.09, 95% CI 1.85–2.37), having been forced for sex in the last 12 months (OR 4.36, 95% CI 3.78–5.03), presence of at least one self reported STI symptom currently (OR 3.50, 95% CI 3.08–3.98) and in the past 12 months (OR 3.58, 95% CI 3.12–4.10) were correlated with HAS. There were no significant differences in the laboratory confirmed STI prevalence rates between those who reported HAS and those who did not (Data not shown in the table).

### Results of Multivariate Analysis

The results of the multivariate model showed that the combined typology of FSWs (AOR 2.20, 95% CI 1.64–2.95), being in the profession longer than three years (AOR 1.69, 95% CI 1.44–1.99) and being literate (AOR 1.28, 95% CI 1.10–1.49) were positively correlated with the practice of HAS. Other socio demographic factors such as having no source of income other than sex work, marital status and STI awareness were not independently associated with reported HAS in the multivariate models.

Risk profile such as engagement in sex work with more than 7 partners in the previous week (AOR 1.78, 95% CI 1.47–2.15), frequent consumption of alcohol (AOR 1.21, 95% CI 1.03–1.42), having experienced forced sex in the past year (AOR 2.24, 95% CI 1.87–2.67), experience of condom breakage in the past month (AOR 2.03, 95% CI 1.70–2.42) were significantly correlated with reported HAS practice. In addition, self risk awareness of HIV acquisition (AOR 1.46, 95% CI 1.25–1.71) experience of at least one self reported STI symptom in the last 12 months, (AOR 1.39, 95% CI 1.13–1.72) and at the time of survey (AOR 1.59, 95% CI 1.30–1.94) were also positively correlated with HAS practice in the FSWs. Although practicing sex work for more than 3 days a week was positively associated with HAS in bivariate analysis (OR 1.15, 95% CI 1.01–1.30), multivariate analysis showed an inverse relationship (AOR 0.78, 95% CI 0.64–0.94). Condom use at last sex with paying partner demonstrated an odds ratio (OR) of 0.85 and reached significance. However, the adjusted odds ratio (AOR) of 0.97 didn’t reach the level of statistical significance. However both OR and AOR were in the same direction.

The Hosmer-Lemeshow Goodness of Fit statistic indicated good model fit [χ^2^ = 12.422, p = 0.133]. The model showed no significant collinearity, multicollinearity (variance inflation factor: VIF<3) and interactions between the predictors examined.

## Discussion

HIV prevalence among FSW participants in our study was 14%. This was higher than the 4.9% prevalence among FSWs reported by National HIV Sentinel Surveillance [HSS] program in India during the comparable period of 2006. [24] The primary reason for this could be that our study was restricted to high HIV prevalence states and HSS results reflect the average national scenario.

### Heterosexual Anal Sex and Condom Use

Twelve percent of our study population of FSWs reported practicing anal sex and among them, condom use at the last anal sex was 73%. It is noteworthy that another study from India on FSWs reported exactly the same prevalence of HAS as in our study. [25] Report of higher prevalence of HAS in FSWs and other high-risk population from India and other countries may suggest possible under-reporting in our survey. [3], [14], [26], [27], [28], [29], [30], [31] A small qualitative study from India has reported that practice of anal sex among FSWs is as high as one third of all sexual encounters. [17] It is possible that large scale quantitative survey inquiring into many aspects of risk behavior may not have accurately elicited reporting of HAS in Indian FSWs. Nevertheless, our study covering four states of India is a large well-planned and well-executed study. Other studies from India from single HIV high prevalent state such as Andhra and Karnataka too have reported findings similar to ours. [32], [33] Despite assumption of under-reporting of HAS, possibly due to cultural taboo and socially desirable response, we clearly document the practice of anal sex in the commercial sex settings which potentially increases the risk of HIV acquisition among FSWs. Our evidence makes a case for including HIV prevention strategies directed at the high-risk practice of HAS by FSWs in the national AIDS prevention and control efforts.

The risk of male-to-female transmission of HIV from unprotected anal sex acts could be very high. [4], [34] In addition, receptive anal sex is a risk factor for STIs, hepatitis B, and Human Papilloma Virus (HPV) related anal cancer. [10], [11], [19], [35], [36], [37] In this study, FSWs reported higher condom use during last anal sex than that reported by high risk and low risk women in other studies from developed and developing countries. [10], [16], [18], [29], [31], [34], [38], [39], [40] Although this is a positive behavior, the possibility of social desirability bias in reporting a perceived stigmatized behavior will have to be kept in mind. Perhaps more confidential types of data collection methods which offer more privacy and anonymity such as Audio Computer Assisted Self Interviewing (ACASI), Informal Confidential Voting Interviews (ICVI) or anonymous envelope technique could have elicited more realistic data on engagement in HAS and condom use at HAS. However, interventions focusing on anal sex and consistent condom use during anal sex might help in reduction of STIs including HIV in the core transmitter group of FSW.

### Literacy and Awareness

The study observation that HAS was more commonly practiced by literate FSWs and those who were aware of their own risk of HIV acquisition suggests that FSWs even from high HIV prevalence states of India may not be fully aware of risk of STI transmission during anal sex. [17] Alternatively they preferred to take a calculated risk by practicing HAS to avoid pregnancy or to earn extra money. [16], [17], [24], [35], [41] Hence prevention messages must stress the higher risk associated with HAS and the importance of use of condoms during both vaginal and anal sex.

### Risk Factors Related to Sex Work

Factors related to the nature of their occupation such as inconsistent use of condom with partners, number of clients entertained and number of days engaged in sex work in a week, forced sex, etc have been found in other studies to be associated with other high risk behavior of FSWs. [14], [16], [19], [25], [30], [41], [42] In this study use of condom at last sex with paying client was significantly associated with reduced report of HAS in bivariate analysis but it did not attain significance in multivariate analysis. However literature reports that consistent condom use by FSWs with their clients encouraged low risk behavior including avoiding HAS or practicing safe HAS. [43] Practicing sex work for more than 3 days in the previous week was inversely related (AOR 0.78, 95% CI 0.64–0.94) to HAS in multivariate analysis though it was positively associated with HAS in bivariate analysis (OR 1.15, 95% CI 1.01–1.30). It could be assumed that when a FSW practices sex work for longer duration, her earnings would be higher and hence such FSWs are less likely to offer anal sex even at higher fees. But positive correlation between entertaining larger number of clients in the previous week with anal sex contradicts this assumption. This could indicate that higher income may not be an exclusive driving factor in HAS practices among FSWs. This is an important finding that warrants more focused qualitative research to provide further insights that would guide the design of interventions. In addition, practice of sex work for fewer days with larger number of clients by these FSWs could expose them to higher number of sexual intercourses per day. This could increase the likelihood that at least one of the acts was HAS. Educational messages stressing safe anal sex should target these FSWs who practice sex work for lesser number of days but have large number of clients.

Data show that vulnerability and inherent risk of HIV acquisition in commercial sex workers due to their nature of work is further amplified by HAS practice. Longer duration in sex work was positively associated with HAS. Duration of sex work in this study was derived from current age and age at first sex work. As 74% of the FSWs in the study were over 25 years of age and consequently they had been in the sex trade for longer duration. Other studies from India have also documented that HAS was practiced more by FSWs who were in the profession for a longer time. [14], [25] The practice of HAS is probably demand driven and older FSWs are vulnerable and unable to refuse anal sex as they get fewer clients [14], [17], [44].

Therefore, older FSWs in sex work for longer duration and/or those entertaining larger number of clients must be sensitized to the risk of HIV transmission associated with anal intercourse. They should be encouraged to use condoms by making them easily available.

### STI and HAS

Evidence of association of HIV and other STIs with HAS is unequivocal. This analysis too showed independent association of presence of at least one STI symptom at the time of survey or in the past 12 months with the practice of HAS. Evidence of association of HIV and other STIs with HAS is mixed. Studies among heterosexual women at low risk as well as at high risk of HIV from USA and Africa found significant association of HAS with verbal report of STI or its symptoms. [9], [16], [20], [26], [35], [41], [45], [46] Results from our bivariate analysis revealed that there was no significant difference in the rate of laboratory confirmed STIs between those who reported HAS and those who did not. But studies of high risk women from Africa have reported that the risk of laboratory confirmed HIV and other STIs was higher among those who practiced HAS. [16], [19], [20], [30] Better level of awareness of STI and self risk for HIV acquisition as well as treatment seeking among FSWs in India might explain the difference. The IBBA survey was designed to identify common STIs through laboratory testing and other Reproductive Tract Infections among FSWs could have been missed in our survey despite reported symptoms suggestive of infection. It is also possible that anal STIs such as rectal gonorrhoea, chlamydia, perianal warts, genital molluscum contagiosum and genital scabies reported in studies on Men who have Sex with Men (MSM) elsewhere in the world may have been missed among our study participants who reported HAS because our study was not aimed at capturing these specific clinical and laboratory end-points. [47], [48] For example the survey tool collected information on abnormal vaginal discharge and ulcerative or blister type of lesions specifically in the genital area which could have led to non detection of lesions in the perianal region. More research is required to elucidate the association of HAS with other non HIV STI, especially anal STI, to understand the associated risk. Anal STI might be missed by health care providers especially in case of women patients unless specifically explored. We strongly recommend examination of ano-rectal region of FSWs for evidence of STI even though they identify themselves as heterosexuals practicing peno-vaginal penetrative sex.

### Other Risk Factors and Vulnerability of FSWs

Results of the study show that other occupation related risks such as frequent consumption of alcohol, experience of forced sex and condom breakage are associated with HAS. Other studies from India and other countries too support these findings. [14], [16], [41], [45], [49], [50], [51], [52], [53] Substance use, especially frequent consumption of alcohol becomes part of life style of FSWs either to increase business or to cope with the demands of the profession. [50] Alcohol could also contribute to risky behaviors including HAS. Positive correlation of inability to use condom despite knowledge about its protective role and desire to use during sex highlights the social and economic vulnerability of FSWs resulting in higher risk of HAS. Interpretations about association of these factors with HAS should be made with caution as these were not explored in depth in our study. Moreover our analysis has >80% power to analyze most of the risk factors except literacy level, number of days entertained clients the previous week and condom use with paying partners at last sex. These findings support the need to design interventions that address larger societal and structural factors contributing to the vulnerability of FSWs associated with their life and profession.

Finally, typology of the FSW has an association with HAS. Significantly higher proportion of FSWs belonging to the combined typology as compared to those who were essentially brothel based reported HAS. This might be due to compromised ability of FSWs in smaller towns to negotiate safe HAS due to lack of awareness as interventions may not have reached this population. As this typology of FSWs could be difficult to reach, implementers of interventions would have to devise strategies to access them and this might prove to be a major challenge to the national program.

Heterosexual transmission predisposes women to risk of acquisition of HIV and STI from their male sex partners and FSW are particularly vulnerable. Currently available option of male condom in various national programs for reducing heterosexual male-to-female transmission is dependent on the male partners’ co-operation and initiative. In many settings as in India, women are unable to negotiate condom use due to their economic dependence on their partners and gender inequality. Likewise FSWs due to the nature of their profession and poverty are more likely to accept offers of sex without condom or anal sex for monetary reasons. These facts highlight the need for women controlled prevention options for vulnerable women such as FSWs. In this context, microbicides to be used vaginally or rectally have the potential to be unique user-controlled or self administered options. [54] Moreover as the study shows that anal sex is practiced by FSWs who are literate, aware of their own risk of HIV acquisition, who entertain large number of clients, who are not able to negotiate condom use, who have been physically forced for sex and those who report frequent condom breakage; microbicides would represent a HIV prevention strategy to these FSWs that doesn’t have to be controlled by their sexual partners.

## Limitation of the Study/Analysis

Our study has certain limitations. Interview-based data on a socially stigmatized issue of HAS may have some limitations in terms of its reliability and validity. In order to maintain comparability of data across districts and states, the questionnaire could not be tailored to include all the locally used terminologies. This might have resulted in underreporting of some variables of interest. Some FSW population groups may not have been sampled using time location sampling method due to the inability of finding them at any fixed place at a particular time as well as the secret nature of the group. Moreover, recording only ‘ever having had anal sex’ could have led to underestimation of the risk due to recall bias. On the other hand, the reported 12% may not reflect the recent practice; which might be actually lower due to greater awareness of HIV risk. Non-response to outcome variable could have influenced subsequent description of risk factors since characteristics of those who chose not to answer this question could have been different. The scope of the FSW survey, data of which are used for this analysis, did not allow for recording qualitative data that could have given insight into the practice of HAS and associated risk factors. Information on number and type of partners with whom FSWs had anal sex, amount paid for anal sex service as compared to vaginal sex, recent practice of anal sex, etc could have provided better understanding of the practice of HAS by this high risk population. Anal STI were not specifically investigated and hence might have been missed. Though some of the risk factors, such as literacy level and days entertained clients last week were significantly associated with HAS, these results have to be interpreted with caution, as these risk factors had less than 80% power for comparison. Study with larger sample size would clarify some of the less defined associations in this study beyond doubt.

## Conclusions

Heterosexual anal sex, a high-risk behavior in the context of HIV transmission was being offered and practiced by nearly 12% FSWs in the four high HIV prevalent States of India. Evidence from this study and other available data help to identify various factors associated with HAS. Interventions to empower FSWs, the core transmitters of HIV in India, to adopt safe anal sex behavior must be designed and offered to them. An important finding of this study was that FSWs might not necessarily be engaging in HAS for additional earnings. This needs to be further qualified through a well-designed qualitative research study. It is also important to openly address the risk of HIV transmission through anal sex and discuss the need for consistent condom use during anal sex as well. Additional efforts too need to be undertaken to reach all non-brothel based sub-groups of FSWs with the same message. Health care providers must be trained to identify anal STIs. Investigations, specifically to rule out anal STI in addition to standard STI tests need to be routinely performed so that HIV acquisition risk due to anal and genital tract STI is reduced. Finally, it is important, not only to prevent HIV transmission from FSWs to their clients, but also to protect them from HIV acquisition. Hence there is an urgent need to develop women controlled options, possibly a microbicide that could be used both vaginally and rectally. It is important to continue efforts to identify a safe rectal microbicide to minimize HIV transmission associated with anal sex.
